# Non-syndromic hearing loss caused by the dominant *cis* mutation R75Q with the recessive mutation V37I of the *GJB2* (*Connexin 26*) gene

**DOI:** 10.1038/emm.2015.32

**Published:** 2015-06-19

**Authors:** Juwon Kim, Jinsei Jung, Min Goo Lee, Jae Young Choi, Kyung-A Lee

**Affiliations:** 1 Department of Laboratory Medicine, Yonsei University Wonju College of Medicine, Wonju, Korea; 2 Department of Otorhinolaryngology, Yonsei University College of Medicine, Seoul, Korea; 3 Department of Pharmacology, Brain Korea 21 Project for Medical Sciences, Severance Biomedical Science Institute, Yonsei University College of Medicine, Seoul, Korea; 4 Department of Laboratory Medicine, Yonsei University College of Medicine, Seoul, Korea

## Abstract

*GJB2* alleles containing two *cis* mutations have been rarely found in non-syndromic hearing loss. Herein, we present a Korean patient with non-syndromic hearing loss caused by the R75Q *cis* mutation with V37I, which arose *de novo* in the father and was inherited by the patient. Biochemical coupling and hemichannel permeability assays were performed after molecular cloning and transfection of HEK293T cells. Student's *t-*tests or analysis of variance followed by Tukey's multiple comparison test was used as statistical analysis. Biochemical coupling was significantly reduced in connexin 26 (Cx26)-R75Q- and Cx26-V37I-transfected cells, with greater extent in Cx26-R75Q and Cx26-R75Q+V37I cells. Interestingly, our patient and his father with the mutations had more residual hearing compared with patients with the dominant mutation alone. Although the difference in hemichannel activity between R75Q alone and R75Q in combination with V37I failed to reach significance, it is of note that there is a possibility that V37I located upstream of R75Q might have the ability to ameliorate R75Q expression. Our study emphasizes the importance of *cis* mutations with R75Q, as the gene effect of R75Q can be modulated depending on the type of additional mutation.

## Introduction

About 50% of autosomal recessive non-syndromic hearing loss cases were reported to be attributed to mutations in the *GJB2* gene (DFNB1; GenBank M86849, MIM121011;13q11-12), which encodes the gap junction protein connexin 26 (Cx26).^1, 2^ This gap junction protein assembles to form channels to allow inorganic ions and small molecules with molecular weights of <1 kDa to pass between adjacent cells.^3^ It is thought to have a significant role in K+ homeostasis and intracellular signaling within the organ of Corti.^4^

The genetic counseling in deaf individuals harboring *GJB2* mutations should be performed with caution as interpretation of mutations and prediction of protein function may be complicated owing to heterogeneity of the gene expression. The severity of hearing loss due to *GJB2* is extremely variable and sometimes cannot be predicted.^5, 6^ The V37I mutation was reported as a non-pathogenic variant, but recent reports suggested that it causes a milder form of hearing loss, which was also confirmed by biochemical and electrophysiological studies.^7, 8, 9, 10^ Additional variants V27I and E114G were also evaluated and found to cause hearing loss in certain conditions, including VG homozygotes or a compound heterozygote with VG.^11^ Interestingly, defects in hemichannel activities were less severe when both loci were mutated, indicating that V27I compensated for the deleterious effect of E114G. Another interesting case showed that R75Q, a dominant *GJB2* mutation, was silenced by the *cis* recessive mutation c.35delG.^12^ Herein, we present a Korean patient with non-syndromic hearing loss caused by the *cis* R75Q mutation with V37I, which arose *de novo* in the father and was inherited by the patient.

## Materials and methods

### Patients

**Figure 1:**
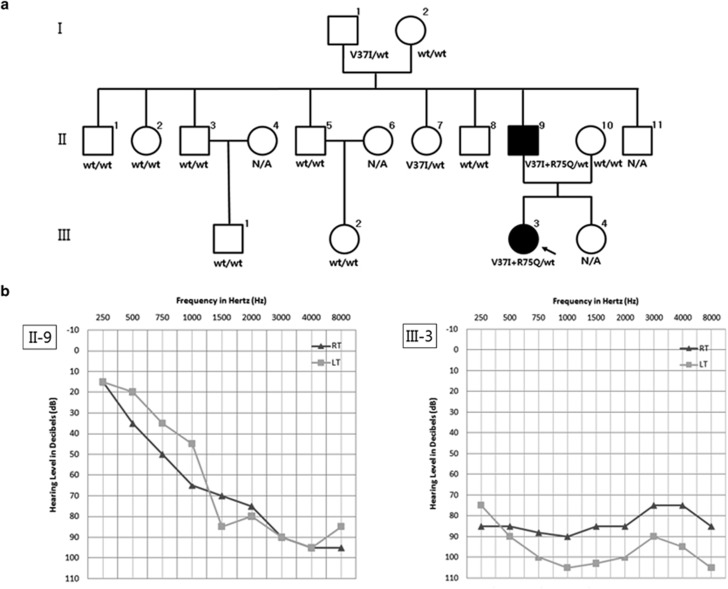
Clinical findings of a patient with non-syndromic hearing loss. (**a**) Pedigree and genotypes of the family. Filled symbols represent ‘affected' individuals (□, males; ○, females; wt, wild type; N/A, not available). The propositus is indicated by an arrow. Note that the R75Q mutation arose *de novo* in affected family member II-9 and was inherited by III-3. I-1 and II-7 were heterozygous for the V37I mutation. The mutations R75Q and V37I were found to be on the same allele. (**b**) Pure tone audiograms of the affected members (II-9 and III-3) of the family.

The propositus (III-3, 16-year-old) (Figure 1a) was diagnosed with non-syndromic hearing loss at the age of 4 and underwent cochlear implantation. Her father (II-9, 45-year-old) had seven siblings, none with hearing impairment. The propositus had bilateral severe hearing loss at the time of diagnosis, but her father showed milder presentation with profound hearing loss at higher frequencies (Figure 1b). Residual hearing at lower frequencies was observed in both of the patients. No dermatologic anomalies or other symptoms that have been reported in patients with syndromic hearing loss were observed in any of the family members including the patients.

Computed tomography of the temporal bone showed no abnormal findings pertaining to the disease in either patient. The study was approved by the Institutional Review Board of the Severance Hospital (IRB#4-2010-0264) and written informed consent was obtained from the patients before blood sampling for further molecular analysis.

### Molecular analysis

All the coding exons and intron transitions of *GJB2* were amplified by PCR and sequenced using the following primers: (forward 1) 5′-TGGTGTTTGCTCAGGAAGAG-3′, (reverse 1) 5′-TTGTGTAGGTCCACCACAGG-3′, (forward 2) 5′-GCCTACCGGAGACATGAGAA-3′, and (reverse 2) 5′-GGCCTACAGGGGTTTCAAAT-3′. The *SLC26A4* gene was also screened for mutations. PCR was performed on 100 ng of genomic DNA using an AccuPower Premix (Bioneer Co., Daejeon, Korea) under the following amplification conditions: 94 °C for 3 min followed by 50 cycles of 94 °C for 1 min, 62 °C for 10 s and 72 °C for 15 s, and final extension was at 72 °C for 15 min. The PCR products were then purified using a QIAquick Gel Extraction Kit (Qiagen, Düsseldorf, Germany) and directly sequenced using a cycle method with the same primers for PCR and a Big Dye Terminator Cycle Sequencing Ready Reaction Kit (Applied Biosystems, Foster City, CA, USA) with the following conditions: 96 °C for 5 min followed by 24 cycles of 96 °C for 10 s, 50 °C for 5 s and 60 °C for 4 min and final extension at 72 °C for 5 min, in conjunction with an ABI Prism 3500dx automated genetic analyzer (Applied Biosystems). For single-strand DNA PCR, the *GJB2* gene was amplified with the primers forward 5′-ATGGATTGGGGCACGCTGC-3′ and reverse 5′-ACGTACATGAAGGCGGCTTCG-3′. The 458-bp PCR product was inserted into the T-blunt vector (SolGent, Daejeon, Korea). After transformation, a single colony was selected, and conventional nucleotide sequencing was performed.

### Molecular cloning and transfection of HEK293T cells

HEK-293T cells obtained from the Korean Cell Line Bank (Seoul, Korea) were maintained in Dulbecco's modified Eagle's medium-HG (Invitrogen, Carlsbad, CA, USA) supplemented with 10% fetal bovine serum and penicillin (50 IU ml^-1^)/streptomycin (50 μg ml^-1^). The coding region of human Cx26 complementary DNA was subcloned into the pEGFP-N1 mammalian expressible plasmid using BamHI and EcoRI restriction sites. Plasmids expressing Cx26-V37I and Cx26-R75Q mutant proteins were generated using a PCR-based site-directed mutagenesis kit (Stratagene, La Jolla, CA, USA). The following primers were used for Cx26-V37I and Cx26-R75Q mutagenesis, respectively: p.V37I (forward) 5′-TATGATCCTCATTGTGGCTGCAAAGGAGGTG-3′, pV.37I (reverse) 5′-GCAGCCACAATGAGGATCATAATGCGAAAAATG-3′, p.R75Q (forward) 5′-CCACATCCAGCTATGGGCCCTGCAGCT-3′, p.R75Q (reverse) 5′-CCATAGCTGGATGTGGGAGATGGGGAAG-3′. Plasmids were transiently transfected into HEK-293T cells using Lipofectamine Plus Reagent (Invitrogen).

### Biochemical coupling assay and hemichannel permeability assay

Gap junction-mediated biochemical coupling and hemichannel (connexon) permeability was measured as described previously.^13^

### Computational modeling

SWISS-MODEL was used to generate model of GJB2 tertiary structure as previously reported.^14^ Cx26-R75Q and Cx26-V37I+R75Q modelings were based on PDB file 2ZW3 template^15^. Molecular graphics and analyses were performed with the USCF Chimera package (http://www.cgl.ucsf.edu/chimera).

### Statistical analysis

The results of multiple experiments are presented as mean±s.e.m. Statistical analysis was performed using Student's *t-*tests or analysis of variance followed by Tukey's multiple comparison test as appropriate. *P*<0.05 was considered statistically significant (SPSS v.18.0, SPSS Inc., Chicago, IL, USA).

## Results

**Figure 2:**
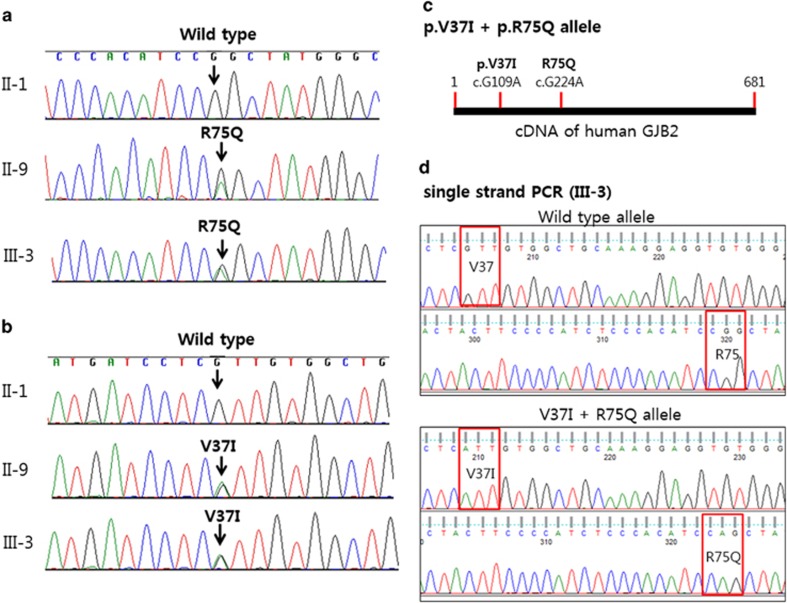
Sequence analysis of the *GJB2* gene. (**a**) Mutations R75Q (c.224G>A; p.Arg75Gln) and (**b**) V37I (c.109G>A; p.Val37Ile) are present in two family members, II-9 and III-3. Both family members are heterozygous for a G to A transition at position 109 and for a transition at position 224. (**c**) V37I and R75Q mutations are depicted in cDNA strand of human GJB2. (**d**) Single-strand PCR chromatograms of III-3 patient are presented. Note that V37I and R75Q are in the same strand.

Molecular analysis of the patients revealed R75Q and V37I mutations in the propositus and her father (Figure 2). No mutation was found in her mother and other family members. Her grandfather (I-1) had V37I but not R75Q mutation, indicating that the R75Q arose *de novo* in the father (II-9) and inherited to the patient III-3. To confirm that dominant mutation R75Q and recessive mutation V37I were presented on the same allele, which is a rare event, single-strand PCR was performed and showed that both mutations were indeed on the same allele (Figure 2c and d). The V37I heterozygous variation was also found in I-1 (grandfather of the propositus) and II-7 (Table 1).

**Figure 3:**
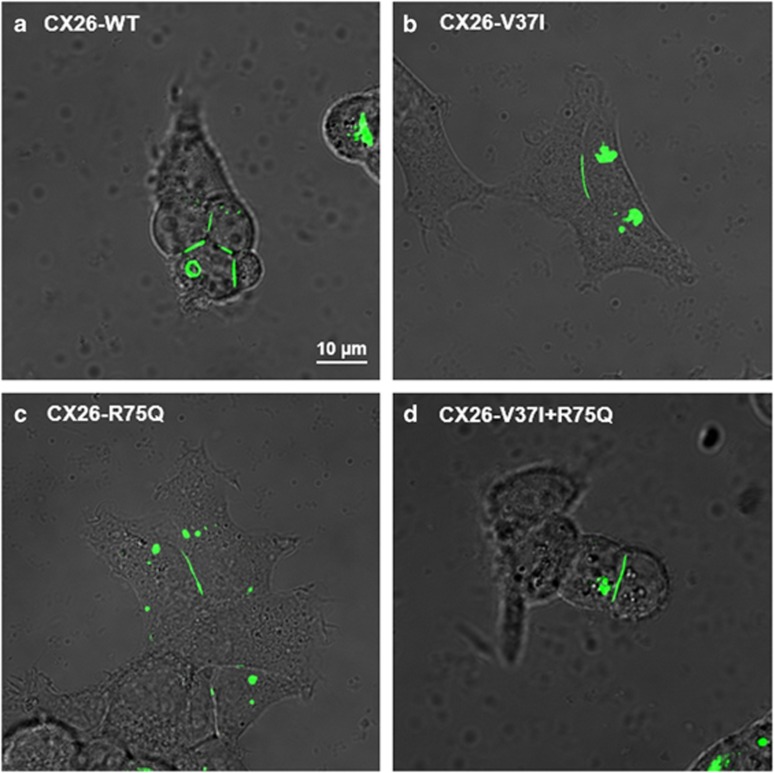
Intercellular localization of the (**a**) Cx26-WT, (**b**) Cx26-V37I, (**c**) Cx26-R75Q and (**d**) Cx26-V37I+R75Q proteins in HEK293 cells. (Scale bar: 10 μm for all panels).

To clarify the possible role of *cis* R75Q and V37I *in vitro* functional analysis was performed. The protein expression and cellular localization of the mutations and wild type (WT) were determined by transfecting HEK293T cells with vectors containing Cx26-WT, Cx26-V37I alone, Cx26-R75Q alone and Cx26-R75Q+V37I (Figure 3). The wild type, as well as the mutant R75Q, V37I, and R75Q+V37I vectors, localized to the plasma membrane at the regions of contact between adjacent cells and formed distinct puncta in the cytoplasm, indicating the ability to form gap junctions at the cell junction.

**Figure 4:**
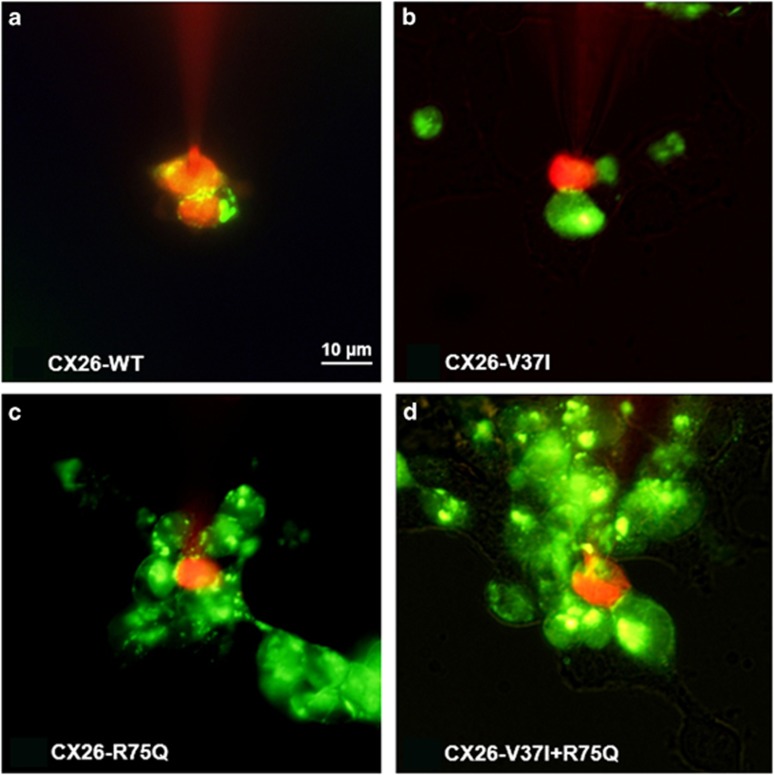
Biochemical coupling through (**a**) Cx26-WT and (**b**–**d**) mutant gap junctions. The intercellular dye transfer using propidium iodide (PI) was conducted. It is of note that, in cells expressing wild-type gap junctions, dye readily traveled through the gap junction to fill the adjacent cells, whereas mutant types showed stasis of the dye in the one cell loaded with PI.

To investigate the biochemical permeability of mutated gap junction channels, cell-to-cell diffusion of propidium iodide (PI) was measured. When PI was injected into the cell, transfer of PI to an adjacent cell was observed in Cx26-WT cells, whereas the Cx26-V37I alone, Cx26-R75Q alone and Cx26-R75Q+V37I cells showed stasis of the dye in one cell (Figure 4). Dye transfer rate was examined by measuring the amount of dye transfer into the adjacent cells and was categorized into fast (transferred <2 min), slow (2–4 min), and no transfer groups (Table 2). The dye transfer rates of Cx26-R75Q and Cx26-V37I+R75Q were markedly reduced such that dye transfer was not observed in 88.2% and 83.3% of the cells examined, respectively. In addition, the dye transfer rate was also reduced in Cx26-V37I alone cells, although less than that of Cx26-R75Q, with a 68.8% rate of no transfer in these cells. The Cx26-WT cells showed efficient dye transfer, and 81.3% of the cells were fast transfer.

**Figure 5:**
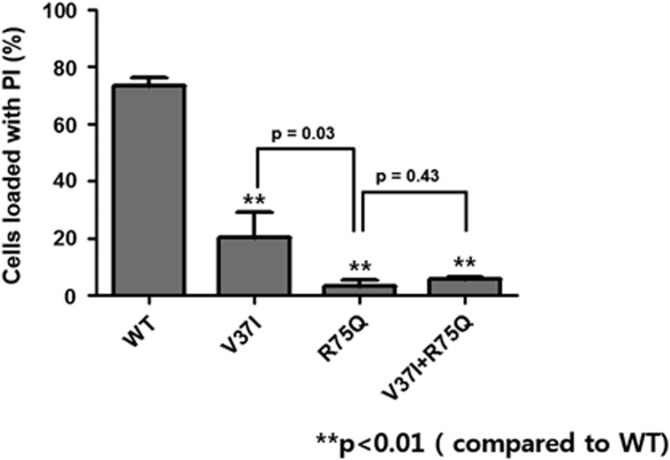
Percent of cells loaded with PI after hemichannel opening induced by the external free Ca^2+^. The PI permeability was significantly reduced in mutant types when compared with the wild type. Each bar represents the mean (±s.e.m.) of at least three images of four different plates of the cells. *P*-value of <0.05 was considered significant.

In the analysis of biochemical permeability of hemichannels, ~92.2% of cells (*n*=1450) transfected with Cx26-WT exhibited PI loading through hemichannels when opened by external free Ca^2+^ solution (Figure 5). On the other hand, 11.2% (*n*=174) of V37I− and 3.34% (*n*=36) of R75Q-transfected cells were loaded with PI after hemichannels were opened by Ca^2+^. The Ca^+2^-induced biochemical permeability of Cx26-R75Q cells was reduced more than that of Cx26-V37I cells, and the difference was statistically significant (*P*=0.03). Co-transfection of Cx26-R75Q and Cx26-V37I had a lower permeability than Cx26-V37I alone but slightly higher than that of Cx26-R75Q alone (*P*=0.43). The Cx26-V37I-, Cx26-R75Q- and Cx26-V37I+R75Q-transfected cells showed significantly reduced PI loading compared with that of Cx26-WT-transfected cells (*P*<0.01).

**Figure 6:**
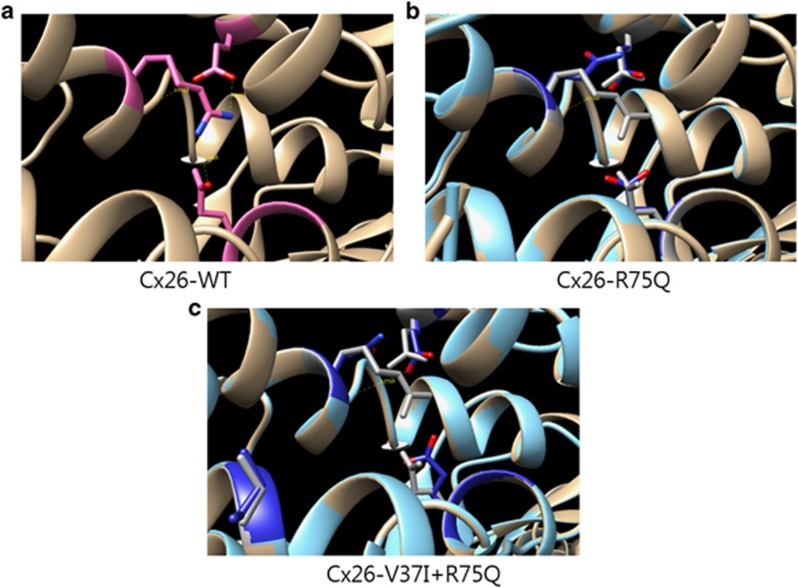
Molecular modeling of Cx26 mutants was performed with 2ZW3 crystal structure template. (**a**) R75 in Cx26-WT interacts with E47, S82 and E187. (**b**) R75Q in Cx26-R75Q has only hydrogen bond with S82. (**c**) The distance of hydrogen bond between R75Q and S82 is farther away and the axis and torsional angle of E47 and E187 are affected by the addition of V37I mutation in Cx26-R75Q.

Finally, we performed computational tertiary structure prediction of GJB2 mutants. 2ZW3 crystal structure was used as template for Cx26 modeling (Figure 6). In Cx26-WT, V37 was located in transmembrane 1 and R75 in transmembrane 2. R75 has hydrogen bonds with E47, S72 (intra-subunit) and E187 (inter-subunit) in Cx26-WT, whereas R75Q did not have hydrogen bonds with E47 and E187. There was no change in the number of hydrogen bonds around R75Q even if V37I mutation was added in Cx26-R75Q. However, the axis and torsion of E47 and E187 were affected by the mutation of V37I. Furthermore, the molecular distance between R75Q and S72 became much farther with the addition of V37I mutation (2.738 Å and 3.276 Å in Cx26-R75Q and Cx26-V37I+R75Q, respectively).

## Discussion

Despite recent progress in the understanding of the genetic basis of hearing loss including the *GJB2* gene, one should be cautious in predicting the effect of a mutation on its function, especially when both mutations are inherited in the same allele. Mutations in *cis* have also been described, albeit infrequently, in other diseases *vis-á-vis* their impact in the development of disease. In cystic fibrosis, the ΔF508/R1158X mutation showed pancreatic insufficiency explained by low stability of truncated protein, whereas the presence of R334Q in *cis* with R1158X conferred mild pancreatic symptoms.^16^ It was predicted that the missense mutation R334Q in *cis* with R1158X somehow stabilized truncated cystic fibrosis transmembrane conductance regulator protein.^16^ On the other hand, the combination of *cis* p.S912L and p.G1244V fibrosis transmembrane conductance regulator protein mutations produced a severe cystic fibrosis phenotype similar to that of p.F508del homozygotes.^17^ In a bilateral moderate hearing loss patient, a dominant mutation R75Q was found in *cis* with the recessive mutation c.35delG, which was thought to be the cause of the disease. However, the family study revealed that the affected sibling of the patient did not have this allele and indicated that R75Q was silenced by c.35delG as c.35delG causes early termination of translation.^12^

Our patient had both the R75Q and V37I mutations in the same allele, which was inherited from her father. To determine the contribution of each mutant to this double-mutant phenotype, we carried out an *in vitro* functional analysis. The R75Q and V37I mutations did not show alteration in the intracellular protein expression study and were localized at the cell membrane and formed gap junctions between two adjacent cells, indicating that the mechanism of hearing loss due to these two mutations does not seem to be pertinent to protein localization. However, biochemical coupling was significantly reduced in Cx26-R75Q- and Cx26-V37I-transfected cells, with the greater extent in Cx26-R75Q- and Cx26-R75Q+V37I-transfected cells. No difference was observed between the cells transfected with Cx26-R75Q+V37I and Cx26-R75Q alone, showing that V37I does not seem to impose significant additional effects in the gap junction function of transfer of ions between the cells.

In the hemichannel study, the number of PI-loaded cells was greatly reduced in Cx26-R75Q- and Cx26-V37I-transfected cells. Although not significant, Cx26-R75Q+V37I-transfected cells displayed a slightly greater percentage of cells with PI loading. These results suggest that the V37I and R75Q mutations significantly reduce hemichannel activity, whereas *cis* mutations slightly improve hemichannel permeability. Glutamine substitution of arginine in Cx26 showed a dominant negative effect (almost null activity) on hemichannel activity as measured by dye loading, consistent with the dominance inheritance of the phenotype. In addition, structural analyses revealed that V37I could impact the structure of Cx26-R75Q (Figure 6). R75Q mutation has dominant negative effect due to the deterioration of hydrogen bond between neighboring subunits of hemichannel (specifically, bond between R75 and E147). Although the deteriorated hydrogen bond was not rescued in the case of Cx26-V37I+R75Q, the change of tertiary structure around R75Q such as longer distance of hydrogen bond between E47 and S72 may affect the quaternary structure and its function of Cx26-V37I+R75Q.

Interestingly, our patients had better residual hearing compared with patients with the dominant mutation alone. Although the difference in hemichannel activity between R75Q alone and R75Q in combination with V37I failed to reach significance, it is of note that there is a possibility that V37I located upstream of R75Q might be able to ameliorate R75Q expression. Future experiments will have to show whether arginine 75 is able to be substituted and whether *cis* V37I somehow induces conformational change of the protein to rescue the R75Q phenotype. In addition to a previous study,^12^ our study emphasizes the importance of *cis* mutations with R75Q as the gene effect of R75Q can be modulated depending on the type of the additional mutation.

## Figures and Tables

**Table 1:** Genotyping by single-strand PCR

*Patient*	*Detected alleles in single strand PCR*
I-1	c.G109A+c.G224/c.G109+c.G224
I-2	c.G109+c.G224
II-9	c.G109A+c.G224A/c.G109+c.G224
II-10	c.G109+c.G224
III-3	c.G109A+c.G224A/c.G109+c.G224

**Table 2:** Percentage of propidium iodide transfer through *GJB2*

*Cx26 type*	*Propidium iodide transfer (%)*		
	*<2 min*	*2~4 min*	*No transfer (%)*
Cx26-WT	13 (82.3)	2 (12.5)	1 (6.3)
Cx26-V37I	2 (12.5)	3 (18.8)	11 (68.8)
Cx26-R75Q	0 (0)	2 (11.8)	15 (88.2)
Cx26-V37I+R75Q	0 (0)	3 (16.7)	15 (83.3)
